# Snap & Write: Examining the Effect of Taking Photos and Notes on Memory for Lecture Content

**DOI:** 10.3390/bs15050561

**Published:** 2025-04-22

**Authors:** Maribeth M. Trego, Julia S. Soares, Annie S. Ditta

**Affiliations:** 1 Department of Psychology, University of California Riverside, Riverside, CA 92507, USA; annie.ditta@ucr.edu; 2 Department of Psychology, Mississippi State University, Mississippi State, MS 39762, USA; js5396@msstate.edu

**Keywords:** photo taking, note taking, education, memory

## Abstract

Three studies investigated the effects of photo and note taking on memory for lecture content in in-person and online environments. Participants watched slideshow lecture videos and were instructed to only watch, take photos, take notes, or simultaneously take both photos and notes of the information on the slides. Memory for on-slide and only said information was tested using fill-in-the-blank questions. Experiment 1, conducted in-person, found a significant photo-taking impairment and an interaction for on-slide information such that there was a larger impairment when participants took both photos and notes compared to when they only took notes. Experiment 2 failed to replicate this interaction; there was an overall photo-taking impairment for on-slide information in an in-person learning environment. We additionally examined mind wandering as a potential mechanism driving these effects but found that it does not provide a sufficient explanation for our results. Experiment 3 used a design similar to Experiments 1 and 2 in an online environment and found a photo-taking benefit for on-slide information. Our results suggest that, in in-person classes, photo taking likely impairs learning, but, in online classes, photo taking may not be as harmful. Participants showed a note taking benefit in Experiments 2 and 3 across both class modalities.

## 1. Introduction

In this technological age, many students take photos of lecture slides instead of or in conjunction with taking notes traditionally. Generally, research has found that note taking improves memory compared to taking no notes (Kiewra, 1989). However, most research shows that photo taking impairs memory of photographed information compared to information that was only observed (Henkel, 2014; Lurie & Westerman, 2021; Niforatos et al., 2017; Soares & Storm, 2018, 2022). That said, the effect of taking photos of lecture slides is less consistent. Some research has found no effect on memory for lectured content when participants took photos of slides compared to when they only watched the lecture, with note-taking resulting in superior memory compared to both (Wong & Lim, 2023). Other work has found a photo-taking benefit for photographed information compared to non-photographed information (Ditta et al., 2023) while others are found the reverse: impaired memory for photographed relative to non-photographed information (Wang et al., 2025). However, students often take photos and notes simultaneously, photographing some slides as they take written notes about those slides and others. Even less is known about the simultaneous effects of photo taking and note taking.

### 1.1. Note Taking

In general, people learn more when they take notes compared to when they do not take notes (Kiewra, 1989; Luo et al., 2018; Mueller & Oppenheimer, 2014). However, this note-taking benefit is moderated by factors such as note quality, quantity, structure, and type of question on the final test. Taking more notes with more factual statements from the learning material is often, but not always, associated with better memory performance (e.g., Kiewra, 1989; Peverly et al., 2013; Mueller & Oppenheimer, 2014; Peverly et al., 2007; Morehead et al., 2019). Taking notes that are paraphrased or summarized also tends to lead to better test performance than taking verbatim notes (Bretzing & Kulhavy, 1979; Bui et al., 2013). Additionally, Peper and Mayer (1986) found that note-taking benefits far-transfer test items significantly more than near-transfer test items. Low-ability participants particularly benefitted from not taking notes when tested for near-transfer (Peper & Meyer, 1978). As such, the effects of note taking can depend on how a student chooses to take notes, what the student is tested on, and the level of knowledge the student has in the subject at the learning phase.

### 1.2. Photo Taking

Taking photos of slides as a note taking strategy constitutes a particularly high-quality, high-quantity note-taking strategy, as taking photos faithfully records all of the information visually presented during a lecture. However, recording such a detailed record may not benefit memory. Given the finding that taking summarized notes is more beneficial than taking verbatim notes (e.g., Bretzing & Kulhavy, 1979), perhaps taking photos of learning material may be unhelpful or even disadvantageous to students since it does not require them to synthesize what they are learning.

Moreover, research on photo taking conducted in contexts outside of the classroom has largely demonstrated a photo-taking impairment such that memory is worse for photographed compared to non-photographed information (Henkel, 2014). The effect has been replicated using smartphone cameras (Niforatos et al., 2017; Soares & Storm, 2018, 2022). The photo-taking impairment effect has been shown using both within-subject designs comparing memory for photographed with non-photographed information and between-subjects designs in which some participants photographed all information while others photographed nothing (Soares & Storm, 2022). The impairment has also been observed on both perceptual and conceptual recognition tests, and on immediate and delayed tests (Lurie & Westerman, 2021).

There are two major proposed mechanisms argued to underlie the photo-taking impairment effect. A cognitive offloading account, first speculatively proposed by Henkel (2014), refers to the idea that photo-takers rely on their cameras to store photographed information for them. As a result, participants fail to effectively encode photographed information compared to non-photographed information. The second major proposed mechanism for the photo-taking impairment effect is that taking a photo causes global attentional disengagement to photographed information (Soares & Storm, 2018). According to this account, participants disengage generally from a photographed scene after they take a photo, and this disengagement persists even once they have put their camera away and are no longer engaging in the dual task of viewing the scene and taking a photo. Consistent with this account, the photo-taking impairment effect has been observed even when participants are given additional viewing time when they take photos to compensate for the time required to operate the camera (Henkel, 2014; Soares & Storm, 2018). An attentional disengagement account predicts that photo taking will impair memory whenever photos are taken, at least so long as attention was paid to the experience prior to taking the photo. A cognitive offloading account instead predicts that photo taking will impair memory only when the photo-taker can rely on the camera to “remember” for them. The photo-taking impairment effect has been shown to persist even when participants know their photos will be deleted and inaccessible (Soares & Storm, 2018), a finding more consistent with attentional disengagement than cognitive offloading.

### 1.3. Effects of Photo Taking on Learning

Though students who take photos of educational material are unlikely to expect that they will have access to their photos on a test, prior work showing the photo-taking impairment even when photos are not expected to be reviewed suggests that similar impairments could emerge when students take photos of lecture slides. However, research on this topic is mixed.

Indeed, Ditta et al. (2023) found a photo-taking *benefit* for online recorded lecture content such that photographed information that was on the slides was remembered significantly better than non-photographed information that was on the slides, even though participants did not have the opportunity to review their photos prior to the test. This benefit was observed regardless of whether participants were instructed to take photos of certain slides or were allowed to choose which slides they wanted to photograph. This finding is consistent with previous work showing that, under some circumstances, taking photos can orient attention to a visual scene (Diehl et al., 2016). However, the photo-taking benefit observed by Ditta et al. (2023) did not always extend to information auditorily presented along with the slides by the lecturer. Prior work suggests that photo taking can have differential effects on memory for auditory and visual information (Barasch et al., 2017), so it is not particularly surprising that photo taking could differentially affect learning of lectured material that appears on-slide compared to information that is only spoken aloud by the lecturer.

Another recent study examined the effect of taking photos during a simulated in-person multimedia lecture on memory when participants did not have the ability to review their photos prior to the test. Contrary to the results of Ditta et al. (2023), Wang et al. (2025) found that participants who watched a lecture and took photos voluntarily had impaired learning compared to participants who only observed the lecture. In Wang et al.’s (2025) second experiment, participants who were assigned to take photos of every slide in a lecture scored lower on a test of their learning than participants assigned to take no photos during the lecture, an effect akin to the photo-taking impairment effect observed between subjects (Soares & Storm, 2022). However, they did not compare memory for on-slide and only-said information specifically.

Other work has also investigated the effects of taking and reviewing photos as an overall “note taking” strategy instead of comparing photographed and non-photographed information. Wong and Lim (2023) compared the effects of taking and reviewing notes on memory using three conditions: longhand note taking only, photo taking only, and a no-note/no-photo baseline. Participants who took written notes had significantly better recall for lecture content compared to participants in the photo taking and baseline conditions. Compared to baseline, participants in the photo taking condition showed no evidence of a photo-taking impairment but also no evidence of a photo-taking benefit, despite the fact that participants who took photos were able to review their photos of every slide. The authors also found that those in the note-taking condition reported less mind wandering than those in the photo taking and control conditions; thus they argued that differences in mind wandering drove the observed note taking benefit.

There is little consensus on the effect of photo taking on memory in a lecture context. That said, the extant studies compared somewhat different factors. Ditta et al. (2023) compared memory for photographed and non-photographed information that appeared in the same lecture, similar to most photo-taking impairment studies. Others compared photo-taking as an overall strategy for recording a lecture to observing the lecture without taking photos (Wang et al., 2025) or to other strategies like traditional note taking (Wong & Lim, 2023).

It is also noteworthy that Wong and Lim (2023) and Wang et al. (2025) conducted their studies in-person, while Ditta et al. (2023) collected data entirely online. There is evidence to suggest that the attentional dynamics differ when participants are in a controlled lab environment versus online. An online participant can choose their own location, and the temptation to multitask or experience interference from outside distractors may be more prevalent online than in a controlled laboratory setting (Wang, 2022). Similar dynamics are likely to occur for remote learners. For example, self-reported attentiveness among students has been shown to vary depending on whether they attend a hybrid, synchronous, or asynchronous online course (Smith & Schreder, 2020). Ditta et al. (2023) speculated that the photo-taking benefit they observed in an online environment could be due to photo taking affecting these attentional dynamics, perhaps by reducing mind wandering or increasing attentional engagement to photographed slides. Specifically, they argued that taking a photo in a completely online context could engage more attention to the lecture compared to not taking a photo, resulting in the photo-taking benefit. As such, differences in learning format could contribute to the mixed results in the literature.

### 1.4. Photo and Note Taking Simultaneously

From an applied perspective, students likely use a combination of strategies, taking notes and photos of lecture slides in tandem, as they attend a lecture. Work on photo taking and its effect on memory for learned material is still developing, and, to our knowledge, no published work has yet investigated how taking both photos and notes simultaneously affects learning. There are many possible outcomes that could result from photo and note taking simultaneously in lectures. If photo and note taking both benefit memory by enhancing attention or engagement, they could have an additive relationship. In this case, taking photos and notes at the same time during a lecture would improve memory more than either method alone. Taking notes at the same time as photos could also decrease mind wandering, since a student would have to focus on managing three tasks (photo taking, note taking, and listening to the lecture) instead of only two, leaving them with little opportunity to mind-wander. However, the task-switching required to take both photos and notes while paying attention to a lecture could also become too cognitively demanding for students to juggle effectively, leading to learning impairments.

Task switching, or switching attention between two or more tasks, has been shown to induce a cost on working memory (Liefooghe et al., 2008), and students could be overwhelmed by the cognitive demands required to juggle two tasks (Draheim et al., 2016). As such, learning impairments might emerge when participants take photos and notes simultaneously that might not be observed when they engage in either task alone. Importantly, this impairment could be observed even if participants report less mind wandering when they engage in both tasks. That is, though students might mind wander less when taking photos and notes simultaneously compared to either strategy alone, the cognitive demands imposed by switching between these tasks might result in a photo-and-note-taking impairment, while engaging in either task alone might cause no such impairment or even memory benefits.

### 1.5. The Current Study

The present work had two main goals. First, it sought to resolve some of the inconsistencies in prior research that investigated the effects of taking photos on educational materials. We did this by comparing memory for photographed and non-photographed information using a design similar to Ditta et al. (2023) in two supervised in-laboratory studies (Experiments 1 and 2) and one online study (Experiment 3). Second, we investigated the effects of simultaneous note and photo taking, both on memory for lecture content and on note effectiveness.

Participants watched recorded PowerPoint lectures, with slides randomly assigned to be viewed only, photographed, viewed while taking notes, or photographed and viewed while taking notes. We used a factorial design such that we could observe the effects of photo taking and note taking separately and their interaction.

Based on various findings in the literature, specifically the conflicting evidence of photo taking effects (or lack thereof) in lecture environments, the robust note-taking benefit effect, and the cognitive costs of task switching, we hypothesized that in Experiment 1, we would find a photo- and note-taking interaction effect such that information that was associated with simultaneous photo- and note-taking would be remembered worse than information that was associated with no strategy or either strategy alone. We also predicted that we would replicate the robust note-taking benefit, but, given the conflicting literature about photo taking in lecture scenarios, we were unsure whether we would observe a photo-taking benefit or impairment.

Across all experiments, as an exploratory measure, we also examined note effectiveness–a construct that encompasses different elements of the notes. Specifically, we examined the effect of simultaneous photo and note taking on note quantity: defined as overall word count; and note quality: defined as the number of correct answers to the final memory test that could be found in the notes. We investigated associations between note effectiveness and memory test performance as well as the differences in note effectiveness between the note-taking and simultaneous photo- and note-taking conditions. We hypothesized that simultaneous photo- and note-taking would result in significantly less effective notes compared to only note-taking, given that engaging in both strategies simultaneously might incur dual-task costs, which result in poorer notes.

## 2. Experiment 1

### 2.1. Participants

A total of 56 undergraduates were recruited from University of California—Riverside participant pool and were given partial course credit for their participation. A power analysis based on Ditta et al. (2023)’s observed effect size of *d* = 0.41 found that 49 participants would be required to detect a similar-sized effect with 80% power and α = 0.05. Data from ten participants were removed for noncompliance with instructions (not taking pictures when asked, *N* = 3; taking notes when not asked, *N* = 7), for a final sample of 46. We determined that this was close enough to our stopping rule to cease data collection. Demographic information was not collected, as we did not have specific hypotheses about how the results would vary based on participant characteristics. However, we expect our sample is reflective of the general participant pool’s demographics. The gender distribution in the pool is 59% female, 40% male, and 0.5% non-binary, and of the undergraduates in the pool, there are 48% freshmen, 26% sophomores, 18% juniors, and 7% seniors.

### 2.2. Design

A fully within-subjects 2 (photo taking: photo vs. no photo) × 2 (note-taking: notes vs. no notes) factorial design was employed. Participants watched two lecture videos under different instructions to create these four conditions. For the photo taking manipulation, participants were instructed to take pictures of a randomly selected half of the lecture slides during both lectures. For the note-taking manipulation, participants were told to take notes throughout one lecture and to take no notes during the other lecture. Performance on a memory test, which included questions about information that appeared on the lectured slides and information that was only spoken aloud by the lecturer, was measured. Notes were also analyzed for effectiveness (i.e., quality and word count).

### 2.3. Materials & Measures

#### 2.3.1. Lecture Videos

Two short lecture videos on cheesemaking and printmaking were used in this study. These topics were chosen because undergraduate psychology students would be unlikely to be familiar with the topics, and they had previously been used by Ditta et al. (2023). These videos consisted of 10 PowerPoint slides containing text and images but no animations and an auditory recording that narrated the slides. Each lecture was approximately 5 min long (range: 4:47–5:23). Each set of materials was adapted from a Wikipedia article on the subjects mentioned above by turning the text into bullet points and adding images. The narration that accompanied the slides was a section of the Wikipedia article about that topic, read in full. On all slides, there was an icon in the bottom left corner that indicated whether participants should take a photo (a camera) or not take a photo of that slide (a camera with a red slash through it).

As described above, there were two lecture types: the photo-only lecture, in which participants took photos of half of the slides and only observed the other half of the slides, and the photo + notes lecture, in which participants took longhand notes in a spiral-bound notebook throughout the lecture while simultaneously taking photos of half of the slides. These two lecture types were then broken down into four conditions for analysis: baseline (slides that were only viewed), photo only (slides that were only photographed), note only (slides for which participants only took notes), and photo + note (slides for which both notes and a photo were taken). The note-taking condition order and assignment to lecture was counterbalanced across participants.

#### 2.3.2. Final Memory Test

Two 15-question fill-in-the-blank tests, one for each of the lectures, were adapted from Ditta et al. (2023). Sample questions include “Cheese is a food derived from milk, produced in a range of flavors, textures, and forms by coagulation of the milk protein ___” (answer: casein); and “In printmaking, each print is thought of as a(n) ___.” (answer: original). These test questions were developed based on the exact text that appeared on the slides and spoken information included in the script and addressed content spread throughout the lecture. All questions for both tests can be found in the Supplementary Materials. Seven questions asked about content that appeared directly on the slides, and eight questions asked about content that was only spoken by the lecturer and did not appear on the slides.

### 2.4. Procedure

The entire study was conducted in the lab using a computer survey built in Qualtrics. After providing informed consent, participants were asked to put their phone on airplane mode, told that they would watch two lecture videos, and would subsequently be tested on the material. Before the photo-only lecture, they were told “*During this lecture, take a photo of each slide that has the following graphic in the bottom left corner*”, (referring to the camera icon), and before the photo + note lecture, they were told “*During this lecture, please take notes on the material while also taking a photo of each slide that has the following graphic in the bottom left corner. You should write your notes in the notebook provided by the researcher. You may format the notes however you like.*”. They were not informed that they would not have a period to review their notes/photos and would not have access to their photos/notes during the test. To familiarize the participants with the photo taking prompts, they were exposed to the photo and no-photo icons before watching the lectures. For the photo taking manipulation, participants used their own smartphones to take the photos. Participants used headphones to listen to the lectures.

Participants then watched the lectures, one for which they took notes and one for which they took no notes. They could not advance to the next part of the study until the full time of the lecture had elapsed. They were watched by an experimenter to ensure that they did not interfere with the lecture (e.g., play the lectures at faster speeds, pause, rewind, or fast-forward the lectures) and that they took photos and notes as instructed. During the note-taking lecture, participants had access to a notebook and pencil in addition to their smartphone. Once the lecture was finished, the notebook and pencil were removed by the researcher.

Once the two lecture videos were complete, students completed an engaging online game, Bubble Shooter, as a distractor task for five minutes. Participants were then prompted to complete the two tests on the lectures and were not allowed to review their notes or their photos before the tests. They were given as much time as needed to complete the tests and were told to only use the knowledge in their head to complete them, so they had no access to their phones, photos, or the internet. The questions for each lecture were presented in a block-randomized order. Within the blocks, the question order was randomized. After completion of both tests, participants were debriefed, granted credit, and thanked for their participation. The entire study took under 30 min to complete.

## 3. Experiment 1 Results and Discussion

### 3.1. Memory Performance

**Figure 1:**
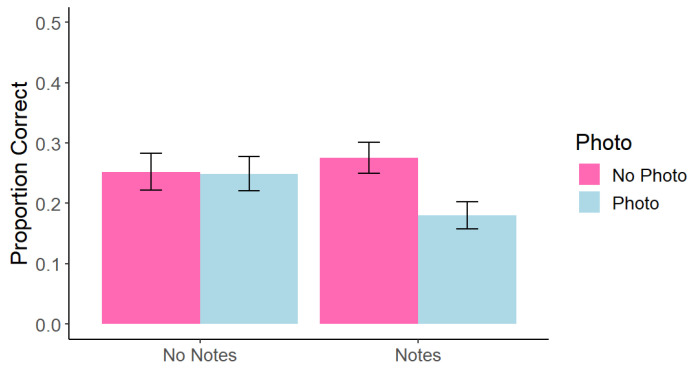
Experiment 1: Correct Answers at Test Across Conditions. Note. Error bars represent standard error.

Memory performance was scored based on the rubric found in the Supplementary Materials. Misspellings were counted as correct. Average scores per condition were calculated to represent a proportion of correct answers out of the total number of questions. A repeated-measures 2 (photo taking: photo vs. no photo) × 2 (note-taking: notes vs. no notes) factorial ANOVA found a significant main effect of photo taking, *F*(1, 45) = 5.93, *p* = 0.020, *η*^2^ = 0.02, such that memory for photographed content (*M* = 0.21, *SD* = 0.14) was impaired relative to memory for non-photographed content (*M* = 0.26, *SD* = 0.16). We did not find a significant main effect of note-taking, *F*(1, 45) = 0.77, *p* = 0.364, *η*^2^ = 0.004 (see Figure 1).

As hypothesized, there was a significant interaction such that there was a larger impairment in the photo + note condition (*M* = 0.18, *SD* = 0.15) compared to the note only condition (*M* = 0.28, *SD* = 0.17), *F*(1, 45) = 4.83, *p* = 0.024, *η*^2^ = 0.02. To further investigate this interaction, we conducted planned comparisons. Focusing on the No Notes condition, there was no evidence of a photo-taking impairment effect *t*(45) = 0.10, *p* = 0.983. However, in the Notes condition, we found a significant photo-taking impairment effect such that performance in the No Photo condition was higher than that in the Photo condition, *t*(45) = 3.97, *p* = 0.001. These results support our hypothesis that participants in the photo + note condition would perform worse compared to the note condition, perhaps due to the high cognitive demand of multitasking between photo- and note-taking, but does not support our hypothesis that participants in the photo + note condition would perform worse compared to the photo-only condition, perhaps due to the beneficial effects of note taking.

**Figure 2:**
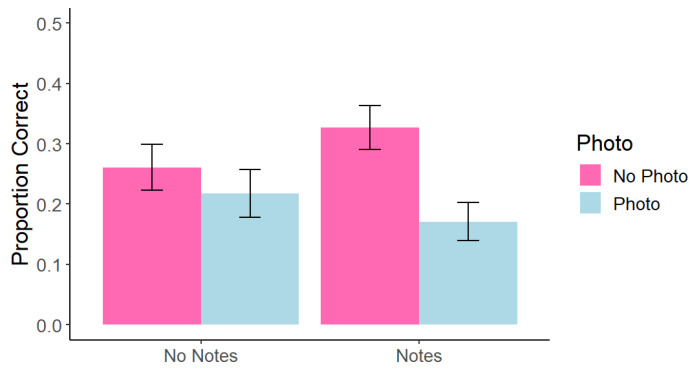
Experiment 1: Correct Answers at Test Across Conditions for On-Slide Information. Note. Error bars represent standard error.

Additionally, like Ditta et al. (2023), we compared test performance separately for information that was presented on the slides (on-slide) and information that was only said by the lecturer (only-said). A repeated-measures 2 (photo taking: photo vs. no photo) × 2 (note taking: notes vs. no notes) factorial ANOVA on the on-slide information found a significant main effect of photo taking, such that memory for photographed on-slide content (*M* = 0.19, *SD* = 0.18) was impaired relative to memory for non-photographed on-slide content (*M* = 0.29, *SD* = 0.14), *F*(1, 45) = 12.37, *p* = 0.001, *η*^2^ = 0.04. We did not find a significant main effect of note-taking, *F*(1, 45) = 0.06, *p* = 0.795, *η*^2^ < 0.001. There was a significant interaction, such that there was a larger impairment in the photo + note condition (*M* = 0.18, *SD* = 0.02) compared to the note only condition (*M* = 0.28, *SD* = 0.03), *F*(1, 45) = 4.23, *p* = 0.046, *η*^2^ = 0.013 (see Figure 2). As in the analysis for the overall memory scores, we found no evidence of a photo-taking effect in the No Notes condition, *t*(45) = 0.98, *p* = 0.760, but a significant photo-taking impairment effect in the Notes condition, *t*(45) = 4.52, *p* = 0.0003. No only-said comparisons were significant; all *p* > 0.076.

### 3.2. Note Effectiveness

We then examined note effectiveness within the note-taking lecture. First, we found that note quantity (i.e., overall word count; *M* = 69.83, *SD* = 28.66) was not significantly correlated with test performance, *r*(44) = −0.04, *p* = 0.777.^1^ Additionally, we examined note quality as the number of correct test answers found in the notes. Specifically, we compared the proportion of answers found in the notes between slides where participants only took notes versus when they were taking photos and notes simultaneously. A paired-samples *t*-test found no significant difference in note quality for the photo + note slides (*M* = 0.16, *SD* = 0.19) and the note only slides (*M* = 0.19, *SD* = 0.16), *t*(45) = −0.98, *p* = 0.330, *d* = 0.12. Additionally, note quality was not significantly correlated with memory performance for either the photo + note slides, *r*(44) = 0.09, *p* = 0.562, or the note only slides (*M* = 0.19, *SD* = 0.16), *r*(44) = −0.06, *p* = 0.695.

The photo-taking impairment found in Experiment 1 when participants took photos and notes simultaneously was consistent with our predictions. It is possible that task switching between taking photos and taking notes may have induced attentional costs beyond just taking notes. Participants may have also disengaged from the main task of learning when they took photos, either during the act of photo taking, or when photographed slides were on the screen.

However, the lack of a note-taking benefit is surprising, given the often-observed benefits of note-taking (Luo et al., 2018; Mueller & Oppenheimer, 2014; Wong & Lim, 2023). The overall photo-taking impairment effect, while driven by the interaction, is in line with past photo taking research in museum contexts (Henkel, 2014), and with one classroom context study (Wang et al., 2025), but misaligned with findings from other learning contexts (Ditta et al., 2023; Wong & Lim, 2023).

## 4. Experiment 2

Given the somewhat contradictory findings across studies finding beneficial, null, and impairment effects of photo taking in lectures, Experiment 2 sought to replicate the effects from Experiment 1 while investigating the mechanism of the photo-taking impairment effect observed. Wong and Lim (2023) claimed that mind wandering mediates the effects of taking photos and notes, so mind wandering probes were included in Experiment 2 to test this mind wandering hypothesis. If mind wandering drives differences in learning effects of photo- and note-taking, we would expect to see the most mind wandering when participants take photos and notes simultaneously, as this condition had the worst memory performance in Experiment 1.

However, taking photos and notes simultaneously is likely the most cognitively demanding condition; if this is the case, then mind wandering rates might instead be lower in this condition compared to others. If this pattern is observed, it would suggest that something other than mind wandering, like increased cognitive demand, drives the photo-taking impairment in the note-taking conditions. Measurements of working memory capacity (WMC) were also included at the end of the study for an exploratory analysis; findings related to WMC can be found in the Supplementary Materials.

### 4.1. Participants

All participants were recruited using the same participant pool as Experiment 1. A total of 237 participated in the study. Data were collected for 2 quarters in an attempt to maximize the probability of finding a decisive result given that Experiment 2 included a close replication of Experiment 1. Data from 21 participants were removed for not following instructions or other technical issues (*N* = 9 for not taking pictures when asked, *N* = 7 for taking notes when told not to, *N* = 4 due to technical issues, and *N* = 1 for requesting their data be removed), for a total of *N* = 216.

### 4.2. Design

The same fully within-subject 2 (photo taking: photo vs. no photo) × 2 (note taking: notes vs. no notes) factorial design from Experiment 1 was employed.

### 4.3. Materials & Measures

All materials and procedural steps were the same as in Experiment 1, with the addition of mind wandering probes and an electronic OSPAN task (described in the Supplementary Materials; Foster et al., 2015).

#### Mind Wandering Probes

Mind wandering was measured using the design from Wong and Lim (2023). A total of four mind wandering probes were included (two per lecture). At two points during each lecture, one at least 30 s into the lecture and one at least 30 s before the ending of each lecture, a chime sound effect was played and the question “Are you mind-wandering?” was presented as an overlay on the slide for two seconds. Participants would then respond with yes or no on a sheet of paper separate from their notes. Participants were informed that mind wandering occurs when one’s attention drifts, and they are no longer fully concentrating on the task at hand. Following the instructions used in Wong and Lim (2023), they were instructed to answer “no” if they were focused on and following the lecture content and/or activities (i.e., note and photo-taking) and “yes” in all other circumstances. No probes were presented during the transition from one slide to another, as participants were likely to be taking photos per the instructions on the new slide that appeared (if applicable).

### 4.4. Procedure

This study was run in Qualtrics with the same instructions and consent process as Experiment 1. However, during the instruction phase, participants were exposed to the mind wandering probe with a practice trial. The practice trial consisted of a 15 s video of a fencing lecture in the same style as the full 5 min lecture videos used in the study, accompanied by the probe overlay. Participants were also informed that their responses about their mind wandering would not affect their participation credit and that they should answer the probe honestly. They were not told how many probes would appear during each lecture.

The lectures and testing were performed following the same procedure as Experiment 1. After the memory test, participants took the electronic OSPAN task (see Supplementary Materials) and were debriefed. The entire study took under 40 min to complete.

## 5. Experiment 2 Results and Discussion

### 5.1. Memory Performance

**Figure 3:**
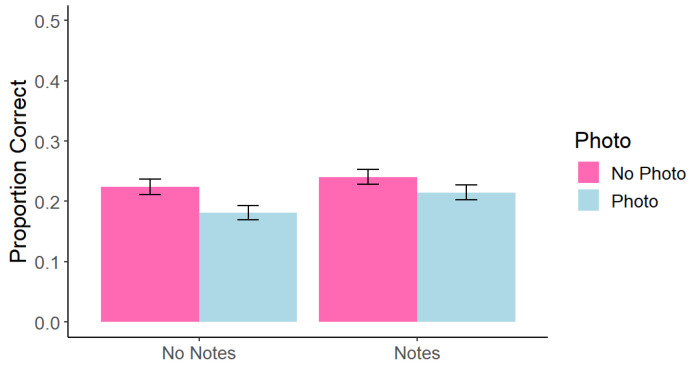
Experiment 2: Correct Answers at Test Across Conditions. Note. Error bars represent standard error.

Test data were scored as in Experiment 1. A within-subjects 2 (photo taking: photo vs. no photo) × 2 (note-taking: notes vs. no notes) factorial ANOVA found a significant main effect of photo taking, *F*(1, 215) = 13.02, *p* < 0.001, *η*^2^ = 0.005, such that memory for photographed content (*M* = 0.20, *SD* = 0.14) was worse than for non-photographed content (*M* = 0.23, *SD* = 0.14). There was also a significant main effect of note-taking such that memory was better when participants took notes (*M* = 0.23, *SD* = 0.16) than when they did not take notes (*M* = 0.20, *SD* = 0.15), *F*(1, 215) = 5.54, *p* = 0.019, *η*^2^ = 0.009. However, we did not replicate the interaction effect from Experiment 1, *F*(1, 215) = 0.57, *p* = 0.452, *η*^2^ < 0.001 (see Figure 3).

**Figure 4:**
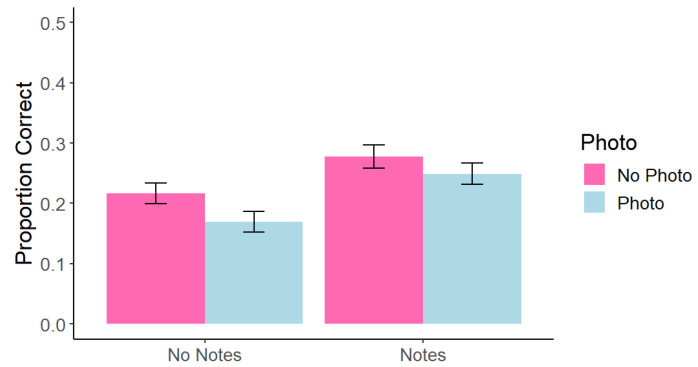
Experiment 2: Correct Answers at Test Across Conditions for On-Slide Information. Note. Error bars represent standard error.

As in Experiment 1, we re-ran the above analysis for on-slide and only-said information separately. We found a significant main effect of photo taking, *F*(1, 215) = 6.06, *p* = 0.015, *η*^2^ = 0.005, such that memory for on-slide photographed content (*M* = 0.20, *SD* = 0.14) was worse than for on-slide non-photographed content (*M* = 0.23, *SD* = 0.14). There was also a significant main effect of note taking, *F*(1, 215) = 18.5, *p* < 0.001, *η*^2^ = 0.02, such that memory for on-slide information was better when participants took notes (*M* = 0.26, *SD* = 0.22) than when they did not take notes (*M* = 0.19, *SD* = 0.20). However, we did not replicate the interaction effect from Experiment 1, *F*(1, 215) = 0.36, *p* = 0.550, *η*^2^ < 0.001 (see Figure 4). No only-said comparisons were significant; all *p* > 0.266.

### 5.2. Mind Wandering

**Figure 5:**
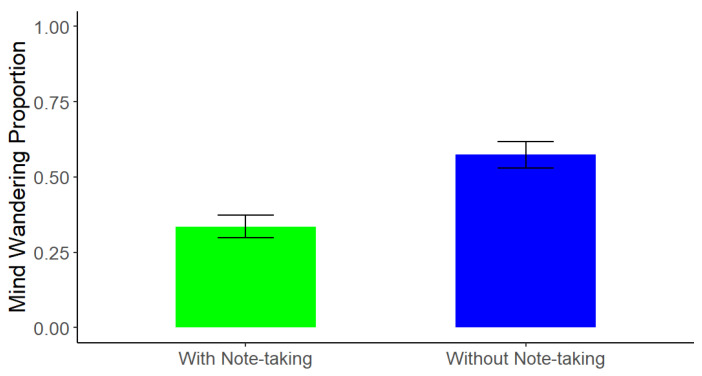
Experiment 2: Mind Wandering Across Lectures. Note. Error bars represent standard error.

Because the design of the study compared memory performance for information on different slides within (photo taking) and between (note taking) the two lectures, the same mind wandering score applied to two conditions each, based on the note-taking manipulation (i.e., the note only and photo + note conditions from the photo + note lecture vs. the photo only and baseline conditions from the photo only lecture). As such, we were not able to directly compare mind wandering as an outcome between all four conditions given that we measured it at the lecture level. Thus, a paired-samples *t*-test on mind wandering rates, ranging from 0.00 (no mind wandering at probe points), to 1.00 (mind wandering at both probe points), between the lecture with note-taking and lecture without note-taking found that participants mind wandered significantly more when they were tasked with only taking photos (*M* = 0.57, *SD* = 0.64) compared to when they were tasked with taking both photos and notes throughout the lecture (*M* = 0.33, *SD* = 0.55), *t*(215) = −4.49, *p* < 0.0001, *d* = 0.40 (see Figure 5). These findings are inconsistent with the idea that mind wandering is the sole mechanism driving the photo-taking impairment effect in lectures, as there were differing levels of mind wandering across the lectures but no evidence of a difference in the size of the memory impairment found when comparing test performance for photographed and non-photographed information across the two note-taking conditions.

### 5.3. Note Effectiveness

**Figure 6:**
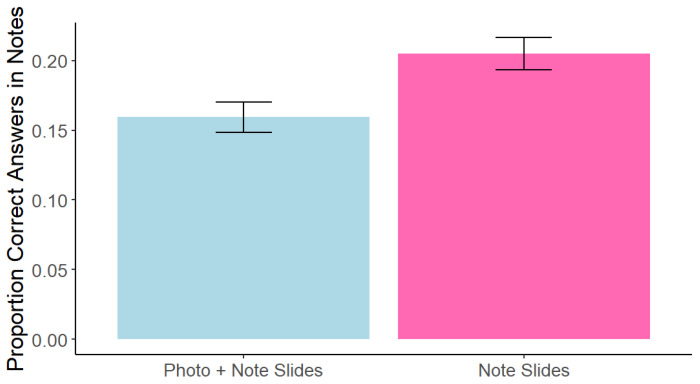
Experiment 2: Note Quality for Notes Taken on Photo + Note vs. Note Slides. Note. Error bars represent standard error.

As in Experiment 1, note effectiveness within the note-taking lecture was examined. Consistent with Experiment 1, overall note quantity (*M* = 70.04, *SD* = 24.27) was not significantly correlated with memory performance, *r*(214) = 1.43, *p* = 0.155. However, in contrast with Experiment 1, a paired-samples *t*-test showed a significant difference in note quality between notes on the photo + note slides (*M* = 0.16, *SD* = 0.16) and the note slides (*M* = 0.20, *SD* = 0.17), *t*(215) = −3.32, *p* = 0.001, *d* = 0.28 (see Figure 6). Note quality was also significantly positively correlated with memory performance for both the photo + note slides, *r*(215) = 0.45, *p* < 0.0001, and the note slides, *r*(215) = 0.50, *p* < 0.0001.

Experiment 2 failed to replicate the photo- and note-taking interaction effect found in Experiment 1, but did replicate the photo-taking impairment effect, in line with Wang et al. (2025)’s findings. However, it remains unclear why the current study demonstrated a photo-taking impairment while Ditta et al. (2023), which used similar materials and procedure, found and replicated a reversal of this pattern. They found a photo taking benefit such that test performance for photographed slides was better than test performance for non-photographed slides, though the effect was more reliable for on-slide compared to only-said information. Notably, Experiments 1 and 2 were both conducted in-person, whereas Ditta et al. (2023) was conducted entirely online.

## 6. Experiment 3

Experiment 3 sought to determine whether an online study context would yield differential photo-taking effects; specifically, we replicated the design of the prior two experiments in an entirely online context. Attention in a remote study might be more susceptible to disruption due to mind wandering, media multitasking, and unexpected interruptions (Smith & Schreder, 2020; Wang, 2022). Thus, in a remote study, asking participants to take a photo of a slide could serve to re-engage their wandering attention on the lecture and confer a benefit not seen in in-person studies where attentional dynamics are different (i.e., participants may already be more focused on the lecture). Thus, we predicted that the photo-taking benefit effect—particularly for on-slide information—would re-emerge in an online context.

Another possibility is that Ditta et al.’s (2023) findings were anomalous. Data from their study were collected during the COVID-19 pandemic from university undergraduates who were largely taking courses online. This could have temporarily changed how these participants interacted with online lectures. Experiment 3 also investigated this possibility; the no note conditions followed Ditta et al.’s (2023) procedure with only small changes.

### 6.1. Participants

All participants were recruited from the same participant pool as the previous two studies. A total of 342 participants were recruited for the study. Due to the participant exclusion rate seen in Ditta et al. (2023), data was collected for 2 quarters to ensure a usable sample. Data from 150 participants were removed for not following instructions (e.g., *N* = 60 for not taking notes when asked or taking notes on both lectures; *N* = 90 for using their notes/photos during the test), for a total of 192 participants included in the analysis. This rate of participant exclusion, while high, is similar to the rate of exclusion observed by Ditta et al. (2023).

### 6.2. Design

The same fully within-subject 2 (photo taking: photo vs. no photo) × 2 (note-taking: notes vs. no notes) factorial design from Experiments 1 and 2 was employed. However, this experiment was run online; participants used personal computers in the location they chose to complete the study. Participants were not supervised, though compliance checks were included to ensure integrity of the data—hence the high participant exclusion rate. Outside distractors were not controlled for as participants were unsupervised; however, this mimics how students engage in remote courses as they must pay attention to the course in the face of many potential distractions.

### 6.3. Materials & Measures

All materials and procedural steps were the same as in Experiment 2, though the OSPAN task was removed because it could only be run using software on an in-lab computer. Additionally, a compliance check was employed to ensure participants’ adherence to the study instructions.

#### Compliance Check

Since researchers were not present to ensure that the study instructions were followed properly, a self-report questionnaire was employed that asked about adherence to photo-, note- and test-taking instructions. Sample questions included “Please type the slide number of each slide you took a photo of in the following boxes. Slide numbers are found in the bottom right of the slide. Please separate each number with a comma. If you do not have the slide numbers in your picture, type the number of slides you took pictures of” (open-ended answer; if participants took less than 4 or more than 5, then they were excluded); and “Did you use your notes or photos to help answer questions during the test?” (answer choices: yes, no). All questions for all tests can be found in the Supplementary Materials. Participants who reported using their notes or photos during the final memory test and those that reported taking no photos or no notes during the specified times were removed from the analysis. Participants were informed that their answers to these questions would not affect their participation credit, so they should answer honestly.

### 6.4. Procedure

The same procedure from Experiment 2 was employed in an online setting. However, since this was an online study and we did not have access to the participants’ physical notes, the participants were asked to transcribe their notes from their notebooks into a provided box on Qualtrics at the end of this study. This allowed us to analyze their note effectiveness as in Experiments 1 and 2. They then completed the compliance check questions directly after completing the memory test but before being debriefed. This entire study took approximately 30 min to complete.

## 7. Experiment 3 Results and Discussion

### 7.1. Memory Performance

**Figure 7:**
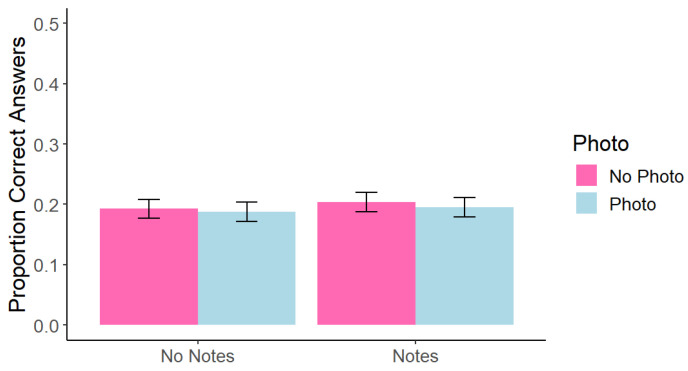
Experiment 3: Correct Answers at Test Across Conditions. Note. Error bars represent standard error.

A within-subjects 2 (photo taking: photo vs. no photo) × 2 (note-taking: notes vs. no notes) factorial ANOVA found no significant main effect of photo taking such that memory for photographed content (*M* = 0.25, *SE* = 0.01) was not significantly different from non-photographed content (*M* = 0.23, *SE* = 0.01), *F*(1, 191) = 3.83, *p* = 0.052, *η*^2^ = 0.003. There was a significant main effect of note-taking such that memory was better for lectures when participants took notes (*M* = 0.26, *SE* = 0.01) compared to when they did not take notes (*M* = 0.22, *SE* = 0.01), *F*(1, 191) = 10.67, *p* = 0.001, *η*^2^ = 0.01. There was no significant interaction, *F*(1, 191) = 0.03, *p* = 0.785, η^2^ < 0.001 (see Figure 7).

**Figure 8:**
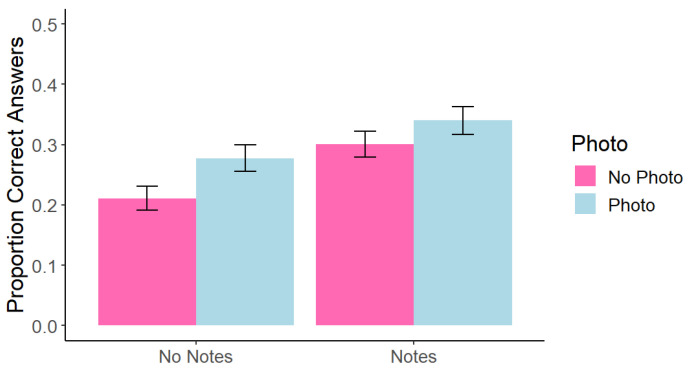
Experiment 3: Correct Answers at Test Across Conditions for On-Slide Information. Note. Error bars represent standard error.

However, as in the previous experiments (and in line with the analyses conducted in Ditta et al., 2023), we analyzed memory performance for the on-slide and only-said information separately. In line with Ditta et al. (2023), a within-subjects 2 (photo taking: photo vs. no photo) × 2 (note-taking: notes vs. no notes) factorial ANOVA found a significant main effect of photo taking, *F*(1, 191) = 6.90, *p* = 0.009, *η*^2^ = 0.008, such that memory for on-slide photographed content (*M* = 0.31, *SE* = 0.002) was better than for on-slide non-photographed content (*M* = 0.26, *SE* = 0.002). There was also a significant main effect of note taking, *F*(1, 191) = 17.06, *p* < 0.001, *η*^2^ = 0.16, such that memory was better when participants took notes on on-slide information (*M* = 0.32, *SE* = 0.02) than when they did not take notes (*M* = 0.24, *SE* = 0.02). However, we again did not replicate the interaction effect from Experiment 1, *F*(1, 191) = 0.57, *p* = 0.451, *η*^2^ < 0.001 (see Figure 8). No only-said comparisons were significant; all *p* > 0.516.

### 7.2. Mind Wandering

**Figure 9:**
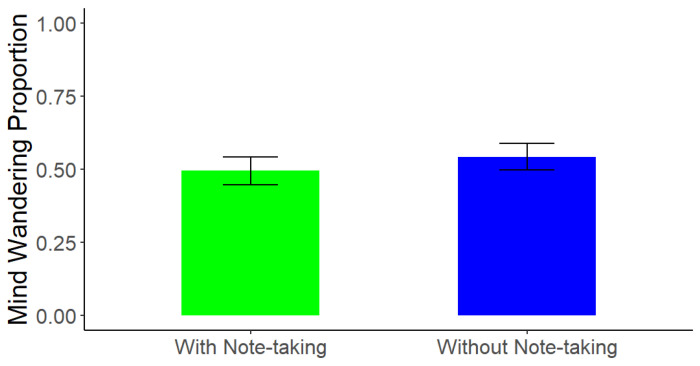
Experiment 3: Mind Wandering Across Lectures. Note. Error bars represent standard error.

A paired-samples *t*-test on mind wandering rates between the lectures did not find a significant difference between when they were only taking photos (*M* = 0.54, *SD* = 0.63) compared to when they were taking photos and notes simultaneously (*M* = 0.49, *SD* = 0.66), *t*(217) = −4.49, *p* = 0.433 (see Figure 9). This is in contrast to Experiment 2, where mind wandering was different across lectures in an in-person context–here we see high levels of mind wandering in both lectures, which is consistent with our proposal that there is less attentional engagement in online environments.

### 7.3. Note Effectiveness

**Figure 10:**
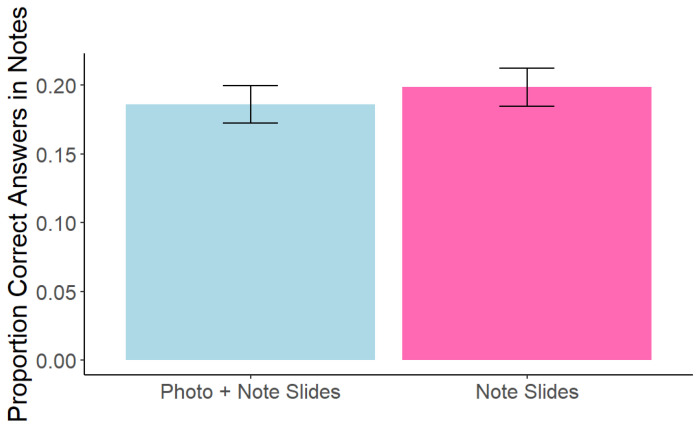
Experiment 3: Note Quality Between Notes Taken on Photo + Note and Note Slides. Note. Error bars represent standard error.

Inconsistent with Experiments 1 and 2, note quantity (*M* = 76.83, *SD* = 55.6) was significantly positively correlated with memory performance, *r*(190) = 0.18, *p* = 0.011. Inconsistent with Experiment 2, a paired-samples *t*-test showed no significant difference in note quality between the photo + note slides (*M* = 0.19, *SD* = 0.19) and the note slides (*M* = 0.20, *SD* = 0.19), *t*(191) = −0.89, *p* = 0.373, *d* = −0.06 (see Figure 10). Note quality was significantly positively correlated with memory performance for both the photo + note slides, *r*(190) = 0.27, *p* = 0.0002, and the note slides, *r*(190) = 0.50, *p* < 0.0001. This means that there was no difference in note quality when taking photos and notes versus only notes and that having high quality notes was associated with better memory regardless of condition. Since note quantity is positively related to performance in an online setting, this could mean that participants who wrote more put more effort and attention towards the experiment. The overall inconsistency in note effectiveness findings across experiments may be due to differing levels of attention or effort in an online study compared to an in-person study.

## 8. General Discussion

In three experiments, we examined how taking photos and notes simultaneously affects memory for lecture content (see Table 1); to our knowledge, this is the first investigation of the effects of this practice on learning. In Experiment 1, we observed a learning impairment associated with simultaneous photo-and note-taking, suggesting that combining these strategies negatively affects memory more than using either strategy alone. However, we failed to replicate this interaction effect in Experiments 2 and 3. In Experiment 2, we found a photo-taking impairment and a note-taking benefit in an in-person learning environment. The effects in Experiments 1 and 2 held when examining memory for on-slide content specifically, though there were no significant effects for only-said information. In Experiment 3, which was conducted in an online context, we found a note-taking benefit, but more importantly, we also found evidence of a photo-taking benefit for on-slide information but not only-said information, replicating the findings of Ditta et al. (2023).

The results of Experiment 1 and 2 align with the majority of past photo taking research that identified a photo-taking impairment effect (e.g., Henkel, 2014; Soares & Storm, 2018) and one study on photo taking in a learning context (Wang et al., 2025). These findings do not align, however, with two previous studies on photo taking in learning contexts (Ditta et al., 2023; Wong & Lim, 2023). However, the results of Experiment 3 align with previous work identifying a photo-taking benefit for on-slide information in an online context (Ditta et al., 2023).

The photo-taking impairment observed in Experiments 1 and 2 could be driven by some factor that is likely to differ across in-person and online settings, like attentional engagement. Participants’ attentional engagement could be higher when watching lectures in a lab setting where they are being monitored compared to participating in an online study where they are not. Indeed, our participant exclusion rates and mind wandering findings may suggest that in-person participants were more engaged than online participants. Our findings from Experiments 1 and 2 suggest that in an in-person learning context, memory for photographed information is impaired relative to non-photographed information. These findings, coupled with those of Wang et al. (2025), suggest that, overall, taking photos in in-person settings may be more harmful than helpful for learning. The photo-taking benefit for on-slide information presented online from Experiment 3 and Ditta et al. (2023) suggest that photo taking may be less detrimental for learning in an online environment. Together, these findings preliminarily suggest that the effect of photo taking on memory for lecture content may depend on the environment, with impairments occurring in in-person (i.e., lower distraction learning scenarios) and benefits occurring online (i.e., distractor heavy learning scenarios), potentially due to differences in attentional engagement across learning modalities (Smith & Schreder, 2020; Wang, 2022). However, this possibility is speculative because it is based on cross-experiment comparisons. Future work should more directly address the possibility that the effects of photo taking on learning from lecture slides depend on one or many of the factors that differ between an in-person and remote learning environment.

Experiments 2 and 3 align with the majority of past note-taking research that shows taking notes benefits memory (Peverly et al., 2013; Mueller & Oppenheimer, 2014; Jansen et al., 2017). Previous research has suggested that lowered rates of mind wandering when participants take notes compared to when they take photos or only watch a lecture may cause these memory benefits (Wong & Lim, 2023). In Experiment 2, we replicated Wong and Lim’s (2023) finding that note-taking participants report less mind wandering compared to when they did not take notes. This finding highlights the importance of note taking as a way to direct attention to a learning task (Jansen et al., 2017). However, in Experiment 3, there was no difference in mind wandering between these conditions. This may be because participants in Experiment 3 engaged less attention overall than their in-person counterparts, though this is speculative (a cross-experiment comparison of overall mind wandering rates between in-person and online environments was non-significant).

One of the goals of this work was to investigate mind-wandering as a mechanism underlying photo and note taking effects. Wong and Lim (2023) argued that mind wandering mediated the beneficial effect of note taking on memory, with the note-taking condition having significantly less mind wandering and thus higher memory performance than the photo taking and baseline conditions. Thus, we hypothesized that there would be lower mind wandering in the photo + note lecture compared to the photo-only lecture because participants would be switching between more tasks. Indeed, this is what we found in Experiment 2. However, both conditions showed a similar photo-taking impairment. Thus, increased mind wandering cannot be the sole reason behind the photo-taking impairment effect. However, these mind wandering findings do not rule out the possibility that attention is involved in driving some of these effects. If attention is involved, it was not apparent in our subjective measures of mind wandering, but it could be apparent using other measures.

Our final, exploratory goal of this project was to examine how note effectiveness is affected by photo and note taking, and how memory performance and note effectiveness are related. Interestingly, the pattern of results was different across experiments. Experiment 1 had no significant note effectiveness findings; this could be due to a power issue, as the power needed was based on photo taking, not note effectiveness, effect sizes. For Experiment 2, which had more participants, we found that participants’ note quality was significantly better when they focused only on taking notes compared to when they engaged in simultaneous photo and note taking. This finding aligned with our prediction that less task switching would be associated with higher note quality. However, in Experiment 3, which was online, there was no significant difference in note quality across the note taking and photo + note conditions. This finding could be due to less attention being devoted to the assigned tasks (photo taking, note taking, and watching the lecture) in the online environment, as shown by the numerically, but not significantly, higher overall rates of mind wandering in Experiment 2 compared to Experiment 3 (though again, cross-experiment comparisons should be interpreted with caution). The general inconsistency of note quantity and its relationship to test performance across the three experiments is not surprising, as the quality and content of the notes are more reliably linked to memory performance than quantity (Bretzing & Kulhavy, 1979; Bui et al., 2013). The relationship of note quality and test performance was found in Experiment 2 and 3 but not Experiment 1; this could be due to this study being underpowered for note quality analysis.

In Experiments 2 and 3, we replicated prior note taking literature (Kiewra, 1989; Peverly et al., 2013), such that higher note quality was significantly and positively correlated with better memory. However, note quantity was only significantly related to memory in an online context; this positive correlation may be due to the attentional dynamics at play in an online environment.

## 9. Limitations and Future Work

One of the major limitations of this series of experiments was the design of the conditions. Because we manipulated photo and note taking to be entirely within participants to increase power, it was not possible to make some comparisons that may have been useful. Specifically, we could not make direct comparisons of mind wandering across our four conditions because we measured mind wandering at the lecture level, not the condition level. Future work could remedy this by assigning four separate lectures—each with one strategy—rather than the two we used. Additionally, future work should strive to measure attention directly in an online versus in-person design so direct claims about attention and context can be made.

The current study used similar materials and procedures across experiments and compared to Ditta et al. (2023), finding both evidence of the photo-taking benefits observed by Ditta and colleagues as well as evidence for a photo-taking impairment effect. Together, these findings suggest that differences in methods, materials, or participant pools are not the factors driving discrepancies across the literature regarding the effects of photo taking on learning of online lecture content. Since we did not directly compare photo and note taking in in-person and online learning contexts within a single study, this speculation relies on cross-experiment comparisons. However, the pattern of fundings suggests that one of the many factors that differ between in-lab and remote learning could play a role in determining the direction of photo-taking effects on learning. To address this possibility, future work should manipulate class context within a single study, as well as systematically investigate how factors that differ between these settings like accountability, distraction, and media multitasking interact with the effects of photo taking.

It is also important to note that this paradigm does not completely replicate learning in realistic classroom situations. For example, our paradigm employs an immediate memory test, rather than a delayed test that would be used in most courses. Past photo taking research has shown a persistent photo taking impairment effect from immediate to delayed testing for both factual and conceptual information (Lurie & Westerman, 2021), but that study was conducted using art objects and not educational materials, so perhaps there are differences that may emerge when using a delayed test for educational materials. An additional consideration is that students may use their photos in conjunction with study strategies (summarizing, rewriting notes) in order to prepare for a delayed test, which could negate the photo-taking impairment. According to Wong and Lim (2023) a photo review period does not significantly improve memory on an immediate test for photo taking compared to reviewing notes, but a review period with a delayed test has not been examined in a learning context.

To address these limitations, future in-lab research should seek to more closely replicate real-life synchronous in-person classroom and online learning environments, where distractions, fatigue, and motivation could be different from a lab/recorded video setting; these moderating variables should be explicitly examined. Finally, future work should consider examining the ways in which individual differences between students (e.g., English language learner status, age, academic discipline, education level, etc.) interact with photo and note taking.

## 10. Conclusions

Overall, this was the first study to examine simultaneous photo- and note- taking behaviors in a lecture context and their subsequent effect on memory. Though more work remains to be carried out to fully characterize the simultaneous effects of photo and note taking in the classroom and to understand the underlying mechanisms behind these effects, our results suggest that students should avoid taking photographs of slides in an in-person context; however, if in an online context, photo taking could benefit memory for on-slide information.

## Figures and Tables

**Table 1:** Memory Effects Across Experiments.

Exp	Environment	Photo Overall	Photo On-Slide	Note	Interaction
1	In-person	Detrimental	Detrimental	*n.s.*	Yes
2	In-person	Detrimental	Detrimental	Beneficial	*n.s.*
3	Online	*n.s.*	Beneficial	Beneficial	*n.s.*

*Note*. ‘Photo’ indicates whether a statistically significant effect was found and the direction of the effect for photo taking on memory. ‘Note’ indicates whether a statistically significant effect was found and the direction of the effect for note-taking on memory. Interaction indicates whether a statistically significant effect was found. Environment refers to whether the experiment was conducted in person or online.

## Data Availability

Data for these studies can be found here: https://github.com/tregomary/Snap-and-Write (accessed on 14 February 2025).

## Note

1 Because both the photo + note and note conditions were created by comparing memory performance for information on different slides within the same lecture, the same set of notes applied to both conditions. As such, we were not able to directly compare word count (i.e., note quantity) as an outcome between the photo + note and note conditions.

## Supplementary Materials

The following supporting information can be downloaded at: https://www.mdpi.com/article/10.3390/bs15050561/s1, Test Questions and Answers; Working Memory Snap and Write.

## References

[B1-behavsci-15-00561] Barasch A., Diehl K., Silverman J., Zauberman G. (2017). Photographic memory: The effects of volitional photo taking on memory for visual and auditory aspects of an experience. Psychological Science.

[B2-behavsci-15-00561] Bretzing B. H., Kulhavy R. W. (1979). Notetaking and depth of processing. Contemporary Educational Psychology.

[B3-behavsci-15-00561] Bui D. C., Myerson J., Hale S. (2013). Note-taking with computers: Exploring alternative strategies for improved recall. Journal of Educational Psychology.

[B4-behavsci-15-00561] Diehl K., Zauberman G., Barasch A. (2016). How taking photos increases enjoyment of experiences. Journal of Personality and Social Psychology.

[B5-behavsci-15-00561] Ditta A. S., Soares J. S., Storm B. C. (2023). What happens to memory for lecture content when students take photos of the lecture slides?. Journal of Applied Research in Memory and Cognition.

[B6-behavsci-15-00561] Draheim C., Hicks K. L., Engle R. W. (2016). Combining reaction time and accuracy: The relationship between working memory capacity and task switching as a case example. Perspectives on Psychological Science.

[B7-behavsci-15-00561] Foster J. L., Shipstead Z., Harrison T. L., Hicks K. L., Redick T. S., Engle R. W. (2015). Shortened complex span tasks can reliably measure working memory capacity. Memory & Cognition.

[B8-behavsci-15-00561] Henkel L. A. (2014). Point-and-shoot memories: The influence of taking photos on memory for a museum tour. Psychological Science.

[B9-behavsci-15-00561] Jansen R. S., Lakens D., IJsselsteijn W. A. (2017). An integrative review of the cognitive costs and benefits of note-taking. Educational Research Review.

[B10-behavsci-15-00561] Kiewra K. A. (1989). A review of note-taking: The encoding-storage paradigm and beyond. Educational Psychology Review.

[B11-behavsci-15-00561] Liefooghe B., Barrouillet P., Vandierendonck A., Camos V. (2008). Working memory costs of task switching. Journal of Experimental Psychology: Learning, Memory, and Cognition.

[B12-behavsci-15-00561] Luo L., Kiewra K. A., Flanigan A. E., Peteranetz M. S. (2018). Laptop versus longhand note taking: Effects on lecture notes and achievement. Instructional Science.

[B13-behavsci-15-00561] Lurie R., Westerman D. L. (2021). Photo-taking impairs memory on perceptual and conceptual memory tests. Journal of Applied Research in Memory and Cognition.

[B14-behavsci-15-00561] Morehead K., Dunlosky J., Rawson K. A. (2019). How much mightier is the pen than the keyboard for note-taking? A replication and extension of Mueller and Oppenheimer (2014). Educational Psychology Review.

[B15-behavsci-15-00561] Mueller P. A., Oppenheimer D. M. (2014). The Pen Is Mightier Than the Keyboard: Advantages of Longhand Over Laptop Note Taking. Psychological Science.

[B16-behavsci-15-00561] Niforatos E., Cinel C., Mack C. C., Langheinrich M., Ward G. (2017). Can less be more?: Contrasting limited, unlimited, and automatic picture capture for augmenting memory recall. Proceedings of the ACM on Interactive, Mobile, Wearable and Ubiquitous Technologies.

[B17-behavsci-15-00561] Peper R. J., Mayer R. E. (1978). Note taking as a generative activity. Journal of Educational Psychology.

[B18-behavsci-15-00561] Peper R. J., Mayer R. E. (1986). Generative effects of note-taking during science lectures. Journal of Educational Psychology.

[B19-behavsci-15-00561] Peverly S. T., Ramaswamy V., Brown C., Sumowski J., Alidoost M., Garner J. (2007). What predicts skill in lecture note taking?. Journal of Educational Psychology.

[B20-behavsci-15-00561] Peverly S. T., Vekaria P. C., Reddington L. A., Sumowski J. F., Johnson K. R., Ramsay C. M. (2013). The relationship of handwriting speed, working memory, language comprehension and outlines to lecture note-taking and test-taking among college students. Applied Cognitive Psychology.

[B21-behavsci-15-00561] Smith J., Schreder K. (2020). Are they paying attention, or are they shoe-shopping? Evidence from online learning. International Journal of Multidisciplinary Perspectives in Higher Education.

[B22-behavsci-15-00561] Soares J. S., Storm B. C. (2018). Forget in a flash: A further investigation of the photo-taking-impairment effect. Journal of Applied Research in Memory and Cognition.

[B23-behavsci-15-00561] Soares J. S., Storm B. C. (2022). Does taking multiple photos lead to a photo-taking-impairment effect?. Psychonomic Bulletin & Review.

[B24-behavsci-15-00561] Wang C. (2022). Comprehensively summarizing what distracts students from online learning: A literature review. Human Behavior and Emerging Technologies.

[B25-behavsci-15-00561] Wang C., Li X., Jin H. (2025). Photograph taking in the classroom impairs memory of learned material. Social Behavior and Personality: An International Journal.

[B26-behavsci-15-00561] Wong S. S. H., Lim S. W. H. (2023). Take notes, not photos: Mind-wandering mediates the impact of note-taking strategies on video-recorded lecture learning performance. Journal of Experimental Psychology: Applied.

