# Supplementary material for: Snap & Write: Examining the Effect of Taking Photos and Notes on Memory for Lecture Content
Source: Behav Sci (Basel). 2025 Apr 22;15(5):561. doi: 10.3390/bs15050561 (PMC12109291; doi:10.3390/bs15050561)
Supplement: Supplementary file 1 [file behavsci-15-00561-s001.zip › Snap and Write_ Test Questions and Answers .pdf]

Question/Answer to Photo and Notes in Lecture

CHEESE

C1

Cheese is a food derived from milk produced in a range of flavors, textures, forms by coagulation of the milk protein _______________. **Casein**

C2

The enzyme _______________ is added to milk to cause coagulation in the cheese making process. **Rennet**

C3

Cheeses are sometimes categorized by _______________, but these categories can be vague or indistinct. **Hardness/texture, firmness**

C4

A better way to categorize cheeses is by _______________. **Technique/how it's made, process 1**

C5

Fresh cheeses tend to be soft, spreadable, and have a _______________ flavor. **mild/mellow, light, bland**

C6

Italian _______________ is an example of a whey cheese. **Ricotta**

C7

Stretched-curd cheeses tend to have a _______________ texture. **fibrous/elastic, stretchy**

C8

In some countries, stretched-curd cheeses are sold in decorative _______________.

braids/forms

C9

Blue cheese is made by _______________ cheese with bacteria.

Injecting/inoculating/infusing

C10

Cheese-making molds tend to range from pale _______________ to dark blue. **green**

C11

Washed-rind cheeses are cured in liquid to make the surface amenable to _______________. **bacteria**

C12

Washed-rind cheeses are _______________ relative to other cheeses.

Labor-intensive/hard, difficult

C13

Brining cheeses is commonly done in hot countries because brining acts as a _______________ technique. **Preservation/preservative, curing**

C14

_______________ is an example of a brined cheese. **Feta**

C15

Processed cheese has a consistent texture and melts _______________. **smoothly/ evenly, easily**

PRINTMAKING

P1

In printmaking, each print is thought of as a(n) _______________. **Original**

P2

The most common material to print on is _______________. **paper**

P3

Printmaking screens are typically made of synthetic or _______ fabrics**. silk**

P4

Woodcut is a method of relief printmaking whereby an artist carves an image into the surface of a block of wood, usually using a _______________. **Chisel**

P5

Woodcut requires relatively little _______________ in the printing process. **Pressure/effort**

P6

Intaglio methods have been used to print bank notes and _______________. **Postage stamps/stamps**

P7

Intaglio is a technique in which the ink is applied to _______________ the original surface of the matrix. **Beneath/under, underneath**

P8

Etching is the process of using a(n) _______________ to cut into the unprotected parts of a metal surface. **Acid/melting, strong acid**

P9

Etching impressions can be used _______________ time(s) before the plate starts to wear. **Several hundred/many**

P10

Planographic methods were originally used for mass printing, but are currently used mostly for _______________ production. **Fine art/art, painting**

P11

Lithography typically uses a(n) _______________ plate treated with acid to create grease reservoirs of the image to be printed. **Limestone/stone, lime**

P12

Lithography was originally used to publish _______________ cheaply. **Theatrical works/scripts**

P13

Stencil techniques are used to print designs onto _______________, but are also well-suited to quick work like street art. **textiles/fabrics**

P14

Screen-printing typically uses fabric stretched across a _______________ frame. **Rectangular**

P15

Screen-printing creates prints using a fabric stencil technique by which ink is pushed through a screen, typically with the use of a _______________. **Squeegee**
