# Supplementary material for: Snap & Write: Examining the Effect of Taking Photos and Notes on Memory for Lecture Content
Source: Behav Sci (Basel). 2025 Apr 22;15(5):561. doi: 10.3390/bs15050561 (PMC12109291; doi:10.3390/bs15050561)

Working Memory Task

A shortened electronic Operation Span Task (OSPAN) from Foster et al. (2015) was employed. OSPAN is a test of WMC in which participants remember sequentially presented letters in order while simultaneously solving mental math equations. Participants first engaged in a practice trial where a sequence of letters interleaved with mental math problems would appear, followed by the letter recall screen. The participants' task was to remember the sequence of letters in order while completing the mental math task. The OSPAN task lasted for two blocks of six trials each, with varying sequence lengths from 3 to 7 (depending on their performance) per trial. Participants had to score at least 85% correct on the math portion for their data to be included, to ensure that they were not simply prioritizing memorization of the letters at the expense of the math problems. The task itself lasted approximately 12 to 15 minutes. The final outcome score used in the analysis was calculated by summing the number of letters correctly recalled in the correct order, as it tends to have high reliability (i.e., the partial span score; Conway et al., 2005).

Working Memory Capacity

A linear regression was conducted to evaluate the effects of WMC, photo condition, note condition, and their interactions on test scores. The overall model was statistically significant, $F(7,840)=2.803$, $p < 0.01$, $R2 = 0.02$, indicating that the predictors collectively explained a small but significant proportion of variance in the test scores (See Figure 1). However, none of the individual or interaction effects were significant. This finding is not surprising, as the photo-note interaction effect, which suggested that participants may have cognitive overload, was not replicated.

Figure S1. WMC, Photo, and Notes Conditions on Test Score by Condition

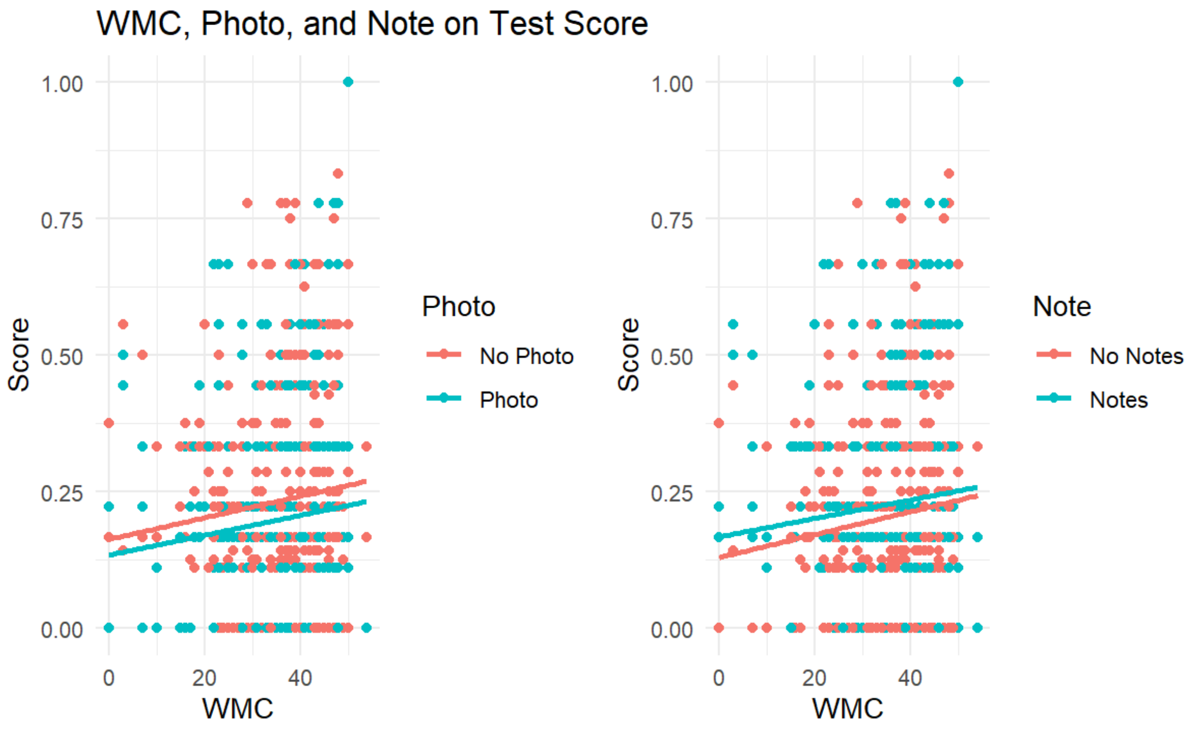

Supplement: Supplementary file 1 [file behavsci-15-00561-s001.zip › Working Memory Snap and Write .pdf]
